# Subjective burden among spousal and adult-child informal caregivers of older adults: results from a longitudinal cohort study

**DOI:** 10.1186/s12877-016-0387-y

**Published:** 2016-12-07

**Authors:** Marloes Oldenkamp, Mariët Hagedoorn, Joris Slaets, Ronald Stolk, Rafael Wittek, Nynke Smidt

**Affiliations:** 1 Department of Epidemiology, University of Groningen, University Medical Center Groningen, PO Box 30.001, 9700 RB Groningen, The Netherlands; 2 Department of Health Sciences, Health Psychology, University of Groningen, University Medical Center Groningen, PO Box 30.001, 9700 RB Groningen, The Netherlands; 3 Department of Geriatrics, University of Groningen, University Medical Center Groningen, PO Box 30.001, 9700 RB Groningen, The Netherlands; 4 Leyden Academy on Vitality and Ageing, Rijnsburgerweg 10, 2333 AA Leiden, The Netherlands; 5 Department of Sociology, University of Groningen, Grote Rozenstraat 31, 9712 TG Groningen, The Netherlands

**Keywords:** Caregiving, Burden, Spouses, Adult children, Longitudinal studies

## Abstract

**Background:**

Pressures on informal caregivers are likely to increase due to increasing life expectancy and health care costs, which stresses the importance of prevention of subjective burden. The present study examined the correlates of overall subjective burden and multiple burden dimensions among spousal and adult-child caregivers of Dutch older adults, both cross-sectional and longitudinal (12-months follow-up).

**Methods:**

In 2010 and 2011 baseline and follow-up data was collected in a sample of informal caregivers and care recipients in the Northern provinces of the Netherlands. Subjective burden included 7 burden dimensions and a summary score for overall subjective burden, based on the Care-Related Quality of Life Instrument (CarerQoL-7D). Objective stressors were the time investment in caregiving (hours of household care, personal care, practical care) and the health situation of the care recipient, including multimorbidity, functional limitations (Katz Index of Independence Basic Activities of Daily Living (ADL) and Instrumental Activities of Daily Living (IADL), and cognitive functioning problems (EQ-5D + C). Correlates of subjective burden were evaluated with linear and logistic regression analyses*.*

**Results:**

The sample consisted of 356 caregivers at baseline (43% spousal, 57% adult-child caregivers), and 158 caregivers at follow-up (45% spousal, 55% adult-child caregivers). At baseline and follow-up, spousal caregivers experienced a higher overall subjective burden, and reported more often mental health problems, physical health problems, and problems with combining daily activities, compared to adult-child caregivers. For spousal caregivers, a poorer health situation of the care recipient was associated with higher subjective burden, while adult-child caregivers reported higher levels of subjective burden when their time investment in caregiving was high. Subjective burden at follow-up was mainly explained by baseline subjective burden.

**Conclusions:**

These results indicate that for effective caregiver support, it is crucial to take the type of care relationship into account, since the level and correlates of overall subjective burden and burden dimensions varied for spousal and adult-child caregivers. In addition, reducing subjective burden will also positively impact the subjective burden over time.

**Electronic supplementary material:**

The online version of this article (doi:10.1186/s12877-016-0387-y) contains supplementary material, which is available to authorized users.

## Background

In many Western countries, informal care is an indispensable part of the health care system. With the recent gains in life expectancy, longer periods of disability, and increasing health care costs, informal care is becoming even more important for our health care system [1]. Pressures on informal caregivers are likely to increase, as expectations and demands placed on them by society will only become higher [2]. In most cases, informal care is directed towards older parents and spouses, with on average 32% of informal caregivers caring for their parent (adult-child caregivers), and 36% caring for their spouse (spousal caregivers) [1]. This caregiving role can be very burdensome, and can even lead to poor health outcomes, such as psychological and physical health problems [3, 4]. Hence, to sustain informal caregiving it is important to prevent excessive burden and promote positive caregiving experiences in informal caregivers.

Within the caregiving population, spousal caregivers are often found to experience higher levels of stress and subjective burden than adult-child caregivers [5, 6]. In a large meta-analysis, Pinquart & Sörensen [5] found that spousal and adult-child caregivers differed on several subjective burden dimensions, but not on overall subjective burden or positive caregiving experiences (‘uplifts of caregiving’) [5]. Specifically, spousal caregivers reported higher levels of physical and financial burden, more depression symptoms, and lower levels of psychological well-being than adult-child caregivers [5]. Furthermore, research showed that objective caregiving stressors like the time investment in caregiving or the care recipient’s health situation, tend to cause higher overall subjective burden [7]. However, the associations of such stressors with subjective burden may differ between spousal and adult-child caregivers [6–8]. For example, care recipient’s physical impairments and behaviour problems had a stronger relationship to subjective burden for spousal caregivers than for adult-child caregivers [7].

With regard to the different dimensions of subjective burden, such as physical or financial burden, there is still much to learn about the associations with objective stressors for both spousal and adult-child caregivers [5, 9]. Better insights in these associations can be highly relevant for caregiver support interventions, as they may be most effective when they address the specific type of burden and associated objective stressor faced by spousal or adult-child caregivers [5]. For example, spousal caregivers often face a high caregiving intensity and suffer from own health problems [5, 6, 10], which might in particular be associated to higher levels of physical burden. Adult-child caregivers, in their turn, are younger and more often combine their informal care tasks with other responsibilities such as paid work [11]. As a result, they may in particular experience problems with managing their multiple tasks.

For the development of effective caregiver support interventions, it is also important to know whether correlates of subjective burden at one time point also determine subjective burden over time. Spousal and adult-child caregivers may benefit from this type of information as it may prevent them from the future development of excessive subjective burden. However, an important drawback of the vast majority of caregiving research is that it concerns cross-sectional research [10]. By studying the correlates of subjective burden both cross-sectionally and over a 12-month period, this study contributes to the current caregiving literature. The main objectives of the study were, firstly, to investigate the extent to which spousal and adult-child caregivers differ in their subjective burden, and secondly, to study the correlates of overall subjective burden and multiple subjective burden dimensions among spousal and adult-child caregivers at one time point (cross-sectional) and over a 12-month period.

## Methods

### Study design

As part of The National Care for the Elderly Programme (Nationaal Programma Ouderenzorg), the Network Elderly Care Region North (Netwerk Ouderenzorg Regio Noord) was set up for the Northern provinces of the Netherlands (Friesland, Groningen, Drenthe, Overijssel). Within this infrastructure, a cohort study was set up to investigate the health care needs of older adults [12]. The Medical Ethical Committee of the University Medical Center Groningen (UMCG) provided a waiver for the cohort study, as it was not an experimental study with test subjects as meant in the Dutch Medical Research Involving Human Subjects Acts (http://www.ccmo.nl/en). Written informed consent was obtained from all participants. Recruitment of participants and baseline data collection took place from April 2010 until January 2011. Follow-up data collection took place from March 2011 until December 2011.

### Recruitment of participants

All organizations in the Network Elderly Care Region North were invited to participate in the study, resulting in 25 participating health care and welfare organizations and elderly associations (i.e. general practitioners, homecare organizations, hospitals, nursing homes). These participating organizations were requested to recruit adults aged 65 years and older from their database. Those with severe cognitive dysfunction, severe physical (terminal) disease(s), or not able to fill out questionnaires, as noted by their physician or caregiver, were excluded from the study. The organizations sent a standardized letter to the older adults, in which the project was explained and informed consent to participate was asked.

Informal caregivers were identified and approached via the participating older adults or the professionals of the health care organization. Informal caregivers were defined as individuals who provided long-term, unpaid care for another individual in their family, household, or social network who has physical, mental, or cognitive limitations [13]. With this broad definition without strict requirements on the intensity and tasks of caregiving or level of impairments of the older adult, we covered the large heterogeneity in the caregiving population. After written consent was given, the caregiver filled out an informal care questionnaire about their caregiving situation and perceptions and experiences of caregiving. In case of multiple caregivers, the caregiver who was most intensively involved in the care was asked to participate. Due to the inclusion procedure, no information was collected about non-response. Follow-up data collection took place on average 12 months after the baseline measurement, and informal caregivers were again invited to fill out the informal care questionnaire.

### Measurements

#### Subjective burden

Subjective burden was measured with the Care-Related Quality of Life Instrument (CarerQoL) [14]. This instrument is comprised of two parts, the CarerQoL-7D and the CarerQoL-visual analogue scale (CarerQoL-VAS), and has shown to have good psychometric properties in several heterogeneous caregiving samples [14, 15]. We used the CarerQoL-7D, which describes care-related subjective burden on seven dimensions, including two positive dimensions (care-related fulfilment and perceived social support) and five negative dimensions (relational problems with the care recipient, mental health problems, problems combining daily activities, financial problems, and physical health problems). Caregivers described their personal situation by responding whether they had no, some, or a lot of problems for each burden dimension. Because of low variation in several dimensions (e.g. 4.8% with a lot mental health problems, 1.1% with a lot of financial problems), we decided to dichotomize the dimensions and combined the categories ‘no’ and ‘some’ for the positive dimension fulfilment (0 = no/some, 1 = a lot) and combined the categories ‘some’ and ‘a lot’ for the other six dimensions (0 = no, 1 = some/a lot). To generate a single summary score based on the seven burden dimensions, a set of weights (a ‘tariff’) was applied to each level of the seven dimensions. This CarerQoL-7D tariff was derived with a discrete choice experiment conducted among the general Dutch adult population [16]. After reverse coding, the CarerQoL-7D summary score represents a score for the overall subjective burden that ranges from 0 (lowest subjective burden) to 100 (highest subjective burden). Because of a moderately positive skewed distribution, we used the square root of the summary score in the statistical analyses (range 0–10) [17].

#### Objective stressors

The *time investment* of caregivers was measured in hours per week and specified for household care, personal care, and practical care tasks. *Household care* included tasks like preparing food and drinks, cleaning the house, or shopping for groceries. *Personal care* concerned, for example, helping with dressing and undressing, washing, toileting, eating, drinking or administering medication. *Practical care* concerned transport, financial, or administrative tasks, such as helping and accompanying with outdoor activities (i.e. family visits, contacts with general practitioner), arranging assistance/devices, or organizing financial and administrative matters. Caregivers were asked to indicate the number of hours for each task, during the past week. This recall method is a valid method to measure time spent on informal care [18]. Nevertheless, when estimating their caregiving time, caregivers might not always take into account the simultaneous performance of multiple caregiving tasks. In addition, they might have difficulties with the distinction between caregiving and non-caregiving related tasks. This could lead them to overestimate their time spent on caregiving [18].

The *health situation of the care recipient* comprised multimorbidity, functional limitations, and cognitive functioning problems of the care recipient. For *multimorbidity*, we used the number of chronic and non-chronic diseases or disorders the care recipient had to deal with during the last year (self-report by care recipient). The list contained 17 diseases or disorders, varying from asthma and broken hip, to diabetes and dementia [19]. Multimorbidity was measured and defined as the total number of diseases or disorders. The degree of *functional limitations* of the care recipient was assessed with the Katz Index of Independence Basic Activities of Daily Living (ADL), and Instrumental Activities of Daily Living (IADL) [20]. Care recipients were asked whether they were dependent on help from others for six basic functions (bathing, dressing, eating, toileting, getting up out of a chair, use of incontinence material), and eight instrumental functions (use of telephone, meal preparation, grooming, travelling, financial management, household tasks, medicine intake, grocery shopping). In addition, care recipients indicated whether they needed assistance while walking [21]. A sum score was computed, with each functional limitation or dependency counting as one. *Cognitive functioning problems* were determined by the single question of the cognitive dimension of the EQ-5D + C (EuroQol-5D + cognitive dimension) [22]. In this question, care recipients were asked whether they had no, some, or severe problems with their memory, concentration, coherence, and/or intelligence.

#### Covariates

Covariates included in the analyses were the caregiver’s age, gender, and self-rated health, the presence of informal support from another informal caregiver or volunteer (no/yes), and whether the care recipient was living in a nursing home or home for the elderly (institutionalized, no/yes), as they have previously been linked to caregiving characteristics and outcomes like objective stressors and subjective burden [10, 23]. Caregiver’s self-rated health was measured with question 1 of the Rand-36: In general, would you say your health is excellent, very good, good, fair, or poor? [24]. Because of low numbers in the category ‘poor’ (0.6%), we combined the categories ‘poor’ and ‘fair’.

#### Statistical methods

The baseline values of spousal and adult-child caregivers were compared, using independent samples *t* tests, Mann–Whitney tests, and Pearson chi-square tests, where appropriate. In addition, the baseline values of the caregivers who dropped out after baseline and the caregivers with scores at both baseline and follow-up, were compared. Uni- and multivariate linear regression analyses (overall subjective burden), and uni- and multivariate logistic regression analyses (subjective burden dimensions), were used to analyse the correlates of subjective burden at baseline and at follow-up, for spousal and adult-child caregivers separately. Independent variables with *p-value* < .10 in the univariate regression analyses were included in the final multivariate regression analyses. For overall subjective burden at follow-up, we first included all independent variables with *p-value* < .10, and subsequently, we added subjective burden at baseline. In case of multicollinearity (condition index >10.0 and/or variance proportions >.50), collinear variables were entered into separate regression models, and presented separately [25]. Listwise deletion was used in all cases (in no case the missing values exceeded 4% per variable). All analyses were performed using IBM SPSS Statistics 22.

## Results

### Study population characteristics

**Fig. 1:**
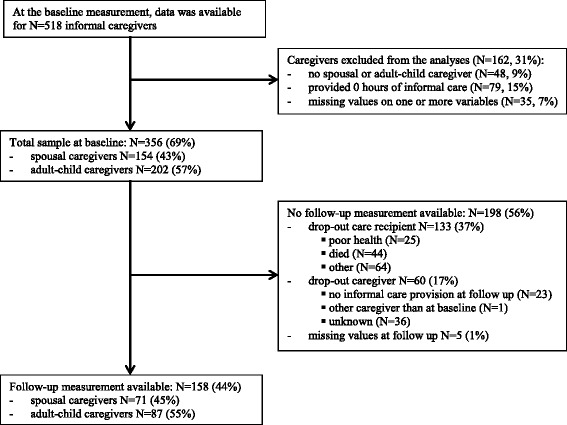
Flow chart of data collection

Between April 2010 and January 2011, 2019 older adults (care recipients) were recruited for participation in the baseline study, and 518 caregivers filled out the informal care questionnaire at baseline (Fig. 1). A total of 356 (69%) out of 518 caregivers, were included in the current study (43% spousal caregivers, 57% adult-child caregivers). At follow-up, only 158 caregivers (44%) still participated (45% spousal caregivers, 55% adult-child caregivers) (Fig. 1). Reasons for lost to follow-up were unknown. Lost to follow-up spousal caregivers did not significantly differ at baseline from spousal caregivers with scores at both baseline and follow-up. Lost to follow-up adult-child caregivers provided on average more hours of household care tasks, and cared more often for a parent (in-law) with more functional limitations or severe cognitive functioning problems, compared to adult-child caregivers with scores at both baseline and follow-up (Additional file 1: Table S1).

Differences between spousal and adult-child caregivers were found for age, gender, self-rated health, hours of household and personal care tasks a week, and the health situation of the care recipient (multimorbidity, functional limitations, and cognitive functioning problems) (Table 1). In addition, spousal caregivers experienced a higher overall subjective burden, and experienced more often mental health problems, physical health problems, and problems with combining daily activities than adult-child caregivers. Spousal caregivers also experienced less often social support.

**Table 1:** Baseline study population characteristics

	All caregivers (*N* = 356)	Spousal caregivers (*N* = 154)	Adult-child caregivers (*N* = 202)	
	Mean (± SD)/N (%)	Mean (± SD)/N (%)	Mean (± SD)/N (%)	*p*
Age CG (40–88)	63.2 (±11.4)	73.3 (± 6.8)	55.4 (± 7.3)	.000
Female CG (N, %)	243 (68%)	90 (58%)	153 (76%)	.001
Type of care relationship (N, %)				
- Spousal CG	154 (43%)			
- Adult-child CG	202 (57%)			
Informal support available (N, %)	124 (35%)	41 (27%)	83 (41%)	.005
Institutionalized CR (N, %)	140 (39%)	14 (9%)	126 (62%)	.000
Self-rated health CG (0–3)	1.2 (±1.0)	.9 (±.9)	1.3 (±1.0)	.000
Time investment – hours a week^a^				
- Household care tasks (0–84)	4.0 (0–12)	12.0 (4–21)	1.5 (0–4)	.000
- Personal care tasks (0–70)	0.0 (0–1)	0.0 (0–4)	0.0 (0–0)	.000
- Practical care tasks (0–56)	2.0 (1–5)	3.0 (1–10)	2.0 (1–4)	.079
Health situation CR				
- Multimorbidity (0–12)	3.4 (± 2.0)	3.0 (± 1.8)	3.6 (± 2.0)	.002
- Functional limitations (0–15)	6.1 (± 4.1)	4.9 (± 4.2)	7.1 (± 3.9)	.000
- Cognitive functioning (N, %)				
- No problems	157 (44%)	74 (48%)	83 (41%)	.040
- Some problems	159 (45%)	70 (46%)	89 (44%)	
- Severe problems	40 (11%)	10 (7%)	30 (15%)	
Subjective burden				
- Overall subjective burden (0–10)	3.9 (±1.7)	4.4 (± 1.5)	3.6 (± 1.7)	.000
- Fulfilment (a lot)	208 (58%)	90 (58%)	118 (58%)	.996
- Relational problems (some/a lot)	126 (35%)	57 (37%)	69 (34%)	.577
- Mental health problems (some/a lot)	149 (42%)	77 (50%)	72 (36%)	.007
- Daily activities problems (some/a lot)	158 (44%)	78 (51%)	80 (40%)	.038
- Financial problems (some/a lot)	22 (6%)	13 (8%)	9 (5%)	.122
- Perceived social support (some/a lot)	229 (64%)	89 (58%)	140 (69%)	.025
- Physical health problems (some/a lot)	177 (50%)	98 (64%)	79 (39%)	.000

*CG* caregiver, *CR* care recipient

^a^Median (interquartile range)

### Correlates of subjective burden at baseline

#### Spousal caregivers

With regard to the overall subjective burden measured at baseline, the results of the multivariate linear regression analyses show that spousal caregivers reported a higher overall subjective burden when they were female, had a poorer self-rated health, and cared for a spouse with more functional limitations or with severe cognitive functioning problems (versus no cognitive functioning problems) (Table 2). The importance of the health situation of the care recipient was, next to being female and a caregiver’s self-rated health, also reflected in the correlates of the different subjective burden dimensions (Additional file 1: Table S2A–C). Spousal caregivers whose spouse had more functional limitations more often experienced relational problems, mental health problems, and problems with combining their daily activities. In addition, a higher multimorbidity was related to more mental health problems, and cognitive functioning problems was related to more relational problems. The time investment in caregiving was neither related to the overall subjective burden of spousal caregivers, nor to the different subjective burden dimensions (Additional file 1: Table S2A–C).

**Table 2:** Linear regression analyses for spousal caregivers (*N* = 154), outcome overall subjective burden at baseline

	Univariate results				Multivariate results			
Spousal caregivers (*N* = 154)	b	(se)	(95% CI)	*p*	b	(se)	(95% CI)	*p*
Constant					**4.177**	(.283)	(3.617; 4.737)	.000
Covariates								
- Age CG	-.004	(.018)	(-.039; .031)	.832				
- Female CG	**.515**	(.241)	(.039; .991)	.034	**.449**	(.221)	(.013; .886)	.044
- Informal support available	.257	(.272)	(-.280; .794)	.346				
- Institutionalized CR	.787	(.414)	(-.031; 1.606)	.059	.245	(.394)	(-.533; 1.023)	.535
- Self-rated health CG	**-.639**	(.117)	(-.870; -.408)	.000	**-.577**	(.116)	(-.806; -.349)	.000
Time investment – hours a week								
- Household care tasks	-.005	(.007)	(-.020; .010)	.507				
- Personal care tasks	.008	(.012)	(-.016; .032)	.513				
- Practical care tasks	.001	(.016)	(-.030; .033)	.932				
Health situation CR								
- Multimorbidity	**.173**	(.065)	(.045; .301)	.008	.101^a^	(.061)	(-.020; .221)	.100
- Functional limitations	**.081**	(.028)	(.026; .137)	.004	**.068** ^a^	(.027)	(.015; .121)	.012
- Cognitive functioning								
- No problems (ref.)								
- Some problems	**.510**	(.242)	(.031; .989)	.037	.380^a^	(.223)	(-.061; .822)	.091
- Severe problems	**1.348**	(.490)	(.380; 2.316)	.007	**.976** ^a^	(.460)	(.068; 1.884)	.035
Adjusted R^2^					.212			

Bold estimates are significant with *p* < .05

*CG* caregiver, *CR* care recipient, *95% CI* 95% confidence interval

^a^results from separate regression models, because multimorbidity, functional limitations, and cognitive functioning were collinear

#### Adult-child caregivers

Considering the overall subjective burden of adult-child caregivers, the results of the multivariate linear regression analyses indicate that adult-child caregivers reported a higher overall subjective burden at baseline when they reported a poorer self-rated health, higher provision of personal care tasks, and when they cared for a parent (in-law) with high multimorbidity (Table 3). With regard to the correlates of the different burden dimensions, we found that the time investment of adult-child caregivers in caregiving (household care and personal care) was mainly associated to the experience of mental and physical health problems, while the health situation of their parent (in-law) was in particular related to the experience of relational problems (Additional file 1: Table S3A-C). Both the time investment in caregiving (personal care tasks) and the health situation of the parent (in-law) (multimorbidity) were related to more problems with combining daily activities.

**Table 3:** Linear regression analyses for adult-child caregivers (*N* = 202), outcome overall subjective burden at baseline

	Univariate results				Multivariate results			
Adult-child caregivers (*N* = 202)	b	(se)	(95% CI)	*p*	b	(se)	(95% CI)	*p*
Constant					**3.651**	(.312)	(3.036; 4.266)	.000
Covariates								
- Age CG	-.005	(.016)	(-.037; .027)	.752				
- Female CG	.287	(.277)	(-.259; .833)	.301				
- Informal support available	-.350	(.241)	(-.824; .124)	.147				
- Institutionalized CR	-.104	(.245)	(-.588; .380)	.672				
- Self-rated health CG	**-.640**	(.110)	(-.858; -.422)	.000	**-.624**	(.108)	(-.837; -.410)	.000
Time investment – hours a week								
- Household care tasks	.035	(.021)	(-.006; .076)	.090	.034^a^	(.019)	(-.003; .071)	.070
- Personal care tasks	**.097**	(.045)	(.008; .186)	.032	**.093** ^a^	(.042)	(.011; .176)	.026
- Practical care tasks	.038	(.026)	(-.014; .090)	.151				
Health situation CR								
- Multimorbidity	**.173**	(.058)	(.060; .287)	.003	**.143** ^b^	(.053)	(.038; .248)	.008
- Functional limitations	.056	(.031)	(-.004; .116)	.069	.048^b^	(.029)	(-.008; .104)	.095
- Cognitive functioning								
- No problems (ref.)								
- Some problems	.311	(.257)	(-.196; .818)	.227				
- Severe problems	.505	(.359)	(-.203; 1.212)	.161				
Adjusted R^2^					.184			

Bold estimates are significant with *p* < .05

*CG* caregiver, *CR* care recipient, *95% CI* 95% confidence interval

^a^results from separate regression models, because household care tasks and personal care tasks were collinear

^b^results from separate regression models, because multimorbidity, functional limitations, and cognitive functioning were collinear

### Correlates of subjective burden at follow-up

#### Spousal caregivers

The results of the multivariate linear regression analysis show that the overall subjective burden of spousal caregivers at follow-up was higher when they cared for an institutionalized care recipient, had a poorer self-rated health at baseline, or were younger (Table 4, note a). By including subjective burden at baseline to the model, which was strongly related to higher subjective burden at follow-up, only age remained statistically significantly related to subjective burden at follow-up (Table 4). Due to the low number of caregivers with follow-up data (71 spousal caregivers, 87 adult-child caregivers), and the low number of cases in cells when combining dependent and independent variables, we were not able to investigate the correlates of the different burden dimensions at follow-up.

**Table 4:** Linear regression analyses for spousal caregivers (*N* = 71), outcome overall subjective burden at follow-up

	Univariate results				Multivariate results^a^			
Spousal caregivers (*N* = 71)	b	(se)	(95% CI)	*p*	b	(se)	(95% CI)	*p*
Constant					**6.531**	(1.618)	(3.298; 9.764)	.000
Covariates								
- Age CG	**-.066**	(.025)	(-.115; -.017)	.009	**-.061**	(.019)	(-.099; -.022)	.003
- Female CG	.118	(.352)	(-.584; .820)	.738				
- Informal support available	.158	(.392)	(-.628; .941)	.688				
- Institutionalized CR	**1.780**	(.645)	(.493; 3.067)	.007	.829	(.552)	(-.273; 1.931)	.138
- Self-rated health CG	**-.709**	(.195)	(-1.099; -.320)	.001	-.307	(.181)	(-.670; .055)	.095
Time investment – hours a week								
- Household care tasks	-.001	(.012)	(-.025; .022)	.917				
- Personal care tasks	-.002	(.019)	(-.040; .035)	.897				
- Practical care tasks	-.027	(.021)	(-.069; .014)	.195				
Health situation CR								
- Multimorbidity	**.270**	(.092)	(.086; .453)	.005	.117	(.082)	(-.046; .281)	.155
- Functional limitations	.031	(.045)	(-.060; .122)	.499				
- Cognitive functioning								
- No problems (ref.)								
- Some problems	**.729**	(.347)	(.038; 1.421)	.039	.420	(.281)	(-.142; .982)	.140
- Severe problems	1.519	(.857)	(-.192; 3.230)	.081	1.031	(.670)	(-.307; 2.369)	.129
Subjective burden at baseline	**.632**	(.115)	(.403; .861)	.000	**.410**	(.120)	(.170; .650)	.001
Adjusted R^2^					.445			

Bold estimates are significant with *p* < .05

*CG* caregiver, *CR* care recipient, *95% CI* 95% confidence interval

^a^If subjective burden at baseline is excluded from the multivariate model, significant estimates are age CG (b -.069, se .021, 95% CI -.111; -.028, p .001), institutionalized CR (b 1.151, se .587, 95% CI -.022; 2.324, p .054), and self-rated health CG (b -.592, se .174, 95% CI -.939; -.244, p .001)

#### Adult-child caregivers

For adult-child caregivers, the results of the multivariate linear regression analysis show that a poorer self-rated health and providing more hours of household care tasks at baseline were related to higher overall subjective burden at follow-up (Table 5, note a). Baseline subjective burden was strongly related to subjective burden at follow-up (Table 5). By adding baseline subjective burden to the model, only the hours of household care tasks at baseline remained statistically significantly related to more subjective burden at follow-up.

**Table 5:** Linear regression analyses for adult-child caregivers (*N* = 87), outcome overall subjective burden at follow-up

	Univariate results				Multivariate results^a^			
Adult-child caregivers (*N* = 87)	b	(se)	(95% CI)	*p*	b	(se)	(95% CI)	*p*
Constant					**1.788**	(.527)	(.739; 2.837)	.001
Covariates								
- Age CG	-.027	(.021)	(-.069; .016)	.214				
- Female CG	**.785**	(.353)	(.082; 1.487)	.029	.411	(.320)	(-.224; 1.047)	.202
- Informal support available	-.099	(.309)	(-.714; .516)	.750				
- Institutionalized CR	.025	(.327)	(-.625; .676)	.938				
- Self-rated health CG	**-.403**	(.148)	(-.696; -.110)	.008	-.144	(.146)	(-.434; .146)	.327
Time investment – hours a week								
- Household care tasks	**.067**	(.023)	(.020; .113)	.005	**.041** ^b^	(.021)	(.000; .083)	.049
- Personal care tasks	.145	(.075)	(-.004; .294)	.056	.042^b^	(.068)	(-.094; .177)	.543
- Practical care tasks	.025	(.031)	(-.037; .087)	.425				
Health situation CR								
- Multimorbidity	.105	(.080)	(-.054; .264)	.192				
- Functional limitations	.058	(.044)	(-.029; .145)	.187				
- Cognitive functioning								
- No problems (ref.)								
- Some problems	.513	(.316)	(-.116; 1.142)	.108				
- Severe problems	.576	(.581)	(-.581; 1.732)	.325				
Subjective burden at baseline	**.498**	(.088)	(.322; .673)	.000	**.414**	(.094)	(.227; .602)	.000
Adjusted R^2^					.295			

Bold estimates are significant with *p* < .05

*CG* caregiver, *CR* care recipient, *95% CI* 95% confidence interval

^a^If subjective burden at baseline is excluded from the multivariate model, significant estimates are self-rated health CG (b -.330, se .154, 95% CI -.637; -.023, p .035) and household care tasks (b .061, se .022, 95% CI .016; .106, p .008)

^b^results from separate regression models, because household care tasks and personal care tasks were collinear

## Discussion

The present study reveals considerable differences between spousal and adult child-caregivers with regard to their care situation and their level of subjective burden. This reaffirms the importance of studying spousal and adult-child caregivers as two separate groups. Spousal caregivers experienced a higher overall burden than adult-child caregivers, as has also been reported in several prior studies [6, 7, 26–28]. The difference in subjective burden was mainly reflected in the finding that spousal caregivers experienced more mental and physical health problems from their caregiving, had more problems with combining their daily activities, and experienced lower social support from their surroundings. However, spousal and adult-child caregivers did not differ with regard to relational problems, financial problems, and fulfilment from caregiving. These results support past arguments that the multidimensionality of subjective burden should be considered, certainly when studying subjective burden among spousal and adult-child caregivers.

Next to differences in subjective burden levels, also the factors related to this subjective burden varied by type of care relationship. Interestingly, for spousal caregivers, the most important correlates of their overall subjective burden and the different burden dimensions were the indicators of the health situation of their care recipient. For adult-child caregivers, their care recipient’s health situation was less important. This supports previous research suggesting that the care recipient’s health situation in particular affects spousal caregivers, because they are most likely living together with their care recipient, and therefore are almost constantly confronted with the health problems of their care recipient [7]. Therefore, caregiver support for spousal caregivers may in particular be effective when it helps them to clear their head now and then, for example by offering them respite care that enables them to take a short break from caregiving. Furthermore, they may benefit from help with dealing with worry and anxiety related to the health problems of their loved one. For adult-child caregivers, the time investment in caregiving appeared to have a greater impact on subjective burden. A higher time investment in caregiving was not only related to a higher overall subjective burden and more mental and physical health problems among adult-child caregivers, but also to more problems with combining their daily activities. These findings correspond with the idea that the time investment in caregiving plays an important role for subjective burden in situations where caregiving has to be combined with more other activities and responsibilities, which is a common situation among adult-child caregivers [29]. Perhaps adult-child caregivers are particularly helped with a reduction in their caregiving tasks and responsibilities, or if that is not possible, with support related to their other activities and responsibilities, such as child care or help with their own household chores.

Remarkably, results showed that, independently from baseline subjective burden, only lower caregiver age predicted burden at follow-up among spousal caregivers, and only household care provision predicted subjective burden at follow up among adult-child caregivers. This supports the idea that most baseline factors affect subjective burden at follow-up indirectly through subjective burden at baseline, for both spousal and adult-child caregivers [9]. It should be noted that we had no information about the caregiving duration, which may have affected our results. Over time, subjective burden may accumulate [30], but caregivers may also be better able to adapt to their caregiving situation [31]. And as suggested in the study of Chappell et al. [9] among Canadian caregivers of older adults recently diagnosed with dementia, this may differ between spousal and adult-child caregivers. They found a higher subjective burden among adult-child caregivers at baseline and at 12 months follow-up compared to spousal caregivers, but only adult-child caregivers, and not spousal caregivers, reported a decrease in burden over time [9]. Future caregiving research will benefit from more longitudinal studies that start shortly after the start of caregiving and cover a longer caregiving period.

In this study, we did not use strict inclusion criteria for participation, for example on type of care or illness of the care recipient, or on the minimal time investment in caregiving. In this way, we intended to capture the heterogeneity of the caregiving population. As a result, our sample may be more representative of the caregiving population in general, compared to studies that examined differences between spousal and adult-child caregivers of people with dementia or other specific health problems [8, 9]. However, our study concerned the Dutch situation, which may limit the generalizability of the findings to other countries. Health care policies and systems differ across countries and may relate to subjective burden of informal caregivers. For example, in a study among 18 European countries, it was found that a generous availability of formal long-term care resources alleviated the negative well-being consequences of informal caregiving [32].

Some other limitations need to be mentioned as well. Firstly, we had no information about reasons for, and percentages of, non-response at baseline among the approached organizations, care recipients, and caregivers. On the one hand, the presence and degree of objective stressors and subjective burden might negatively affect a caregiver’s participation in research [33]. On the other hand, there is also evidence suggesting that highly involved caregivers (i.e. high time investment, high subjective burden) are more likely to participate in caregiving research, and that the influence of non-response on associations between objective stressors and subjective burden is only small [34]. Secondly, loss to follow-up was quite high (56%), which restricted our analyses of the different burden dimensions at follow-up. Spousal caregivers lost to follow up and not lost to follow up did not significantly differ. Because of indications of selective drop-out among adult-child caregivers, we repeated the cross-sectional analyses in the smaller sample including only adult-child caregivers with scores at both measurements (*N* = 87), and compared the results with the original cross-sectional results based on the total sample of adult-child caregivers (*N* = 202). Except for some differences in statistical significance, which can be explained by the lower power in the smaller sample, results were largely comparable in direction and size. However, it should be taken into account that the high level of drop-out may have influenced the study results, and the possibility of selective drop-out on non-observed characteristics still exists. Thirdly, the small number of caregivers with financial problems due to their caregiving responsibilities (6%), restricted our analyses of the correlates of financial burden, and contradicts earlier studies in which financial strain has been found to be evident [27, 35], in particular among spousal caregivers [5]. However, in other studies in which the CarerQoL-7D was used, also only few caregivers reported financial difficulties due to their caregiving responsibilities [14, 15, 36]. Because financial strain due to caregiving can have multiple causes (e.g. reduction in household income, increase in expenditures related to the care and treatment of the care recipient) [37], and can be measured in multiple ways, it is difficult to explain the differences in financial burden between studies. And finally, we were unable to consider possible interactions with roles or obligations in other life domains than the caregiving role, which might in particular be applicable to adult-child caregivers. Incorporating possible spill-overs between roles, such as being caregiver, employee, parent, and sibling, will enhance caregiver research, because subjective burden and quality of life of caregivers are determined by many roles and situations in life, including, but not restricted to, the caregiver role [38].

## Conclusions

Our findings underscore once more that when supporting informal caregivers, the type of care relationship should definitely be taken into account. Spousal and adult-child caregivers provide care in different caregiving situations, may experience different levels and types of burden, and these burdens may be associated to different caregiving stressors. In addition, an important conclusion from our longitudinal study is that the current health situation of the care recipient and the current time investment in caregiving are important predictors of subjective burden over time, and should be taken into account when supporting informal caregivers.

## Additional file

Additional file 1: Table S1. Comparison caregivers dropout after baseline and caregivers with scores at both time periods. **Table S2.** Baseline univariate and multivariate logistic regression analyses, spousal caregivers (*N* = 154). **Table S3.** Baseline univariate and multivariate logistic regression analyses, adult-child caregivers (*N* = 202). (DOCX 52 kb)

## Abbreviations

ADL: Activities of Daily Living
CarerQol: Care-Related Quality of Life Instrument
EQ-5D + C: EuroQoL-5D + cognitive dimension
IADL: Instrumental Activities of Daily Living
VAS: Visual analogue scale
