# Supplementary material for: Subjective burden among spousal and adult-child informal caregivers of older adults: results from a longitudinal cohort study
Source: BMC Geriatr. 2016 Dec 7;16:208. doi: 10.1186/s12877-016-0387-y (PMC5142272; doi:10.1186/s12877-016-0387-y)
Supplement: Additional file 1: Table S1. — Comparison caregivers dropout after baseline and caregivers with scores at both time periods. Table S2. Baseline univariate and multivariate logistic regression analyses, spousal caregivers (N = 154). Table S3. Baseline univariate and multivariate logistic regression analyses, adult-child caregivers (N = 202). (DOCX 52 kb) [file 12877_2016_387_MOESM1_ESM.docx]

Additional file 1: ‘Subjective burden among spousal and adult-child informal caregivers of older adults: results from a longitudinal cohort study’

| Table S1: Comparison caregivers dropout after baseline and caregivers with scores at both time periods | | | | | | |
| --- | --- | --- | --- | --- | --- | --- |
|  | Spousal caregivers (N=154) | | | Adult-child caregivers (N=202) | | |
|  | Mean (± SD) / N (%) | | | Mean (± SD) / N (%) | | |
|  | Drop out (N=83, 54%) | Included (N=71, 46%) | p | Drop out (N=115, 57%) | Included (N=87, 43%) | p |
| Age CG | 73.4 (±6.8) | 72.7 (±6.8) | .282 | 54.7 (±56.4) | 56.4 (±7.2) | .093 |
| Female CG | 49 (59%) | 41 (58%) | .871 | 86 (75%) | 67 (77%) | .714 |
| Type of care relationship  - Spousal CG  - Adult-child CG |  |  |  |  |  |  |
| Informal support available | 22 (27%) | 19 (27%) | .972 | 46 (40%) | 37 (43%) | .718 |
| Institutionalized CR | 9 (11%) | 5 (7%) | .413 | 67 (58%) | 59 (68%) | .165 |
| Self-rated health CG | 1.0 (±1.0) | .8 (±.8) | .242 | 1.3 (±1.0) | 1.4 (±1.0) | .761 |
|  |  |  |  |  |  |  |
| Time investment – hours a week ^a^ |  |  |  |  |  |  |
| - Household care tasks | 12 (3-20) | 12 (6-21) | .725 | 1 (0-4) | 2 (0-6) | .008 |
| - Personal care tasks | 0 (0-4) | 0 (0-4) | .563 | 0 (0-0) | 0 (0-0) | .937 |
| - Practical care tasks | 2 (0-10) | 3 (1-8) | .474 | 2 (1-4) | 2 (1-4) | .913 |
|  |  |  |  |  |  |  |
| Health situation CR |  |  |  |  |  |  |
| - Multimorbidity | 2.9 (±1.9) | 3.1 (±1.8) | .569 | 3.5 (±2.1) | 3.8 (±1.9) | .401 |
| - Functional limitations | 4.6 (±4.5) | 5.2 (±3.4) | .362 | 7.7 (±4.0) | 6.3 (±3.5) | .011 |
| - Cognitive functioning  - No problems  - Some problems  - Severe problems | 45 (54%)  31 (37%)  7 (8%) | 29 (41%)  39 (55%)  3 (4%) | .079 | 46 (40%)  46 (40%)  23 (20%) | 37 (43%)  43 (49%)  7 (8%) | .054 |
|  |  |  |  |  |  |  |
| Subjective burden |  |  |  |  |  |  |
| - summary score | 4.4 (±1.7) | 4.4 (±1.3) | .860 | 3.6 (±1.8) | 3.6 (±1.5) | .871 |
| - fulfilment (a lot) | 52 (63%) | 38 (54%) | .252 | 64 (56%) | 54 (62%) | .360 |
| - relational problems (some/a lot) | 28 (34%) | 29 (41%) | .362 | 38 (33%) | 31 (36%) | .701 |
| - mental health problems (some/a lot) | 45 (54%) | 32 (45%) | .258 | 40 (35%) | 32 (37%) | .769 |
| - daily activities problems (some/a lot) | 42 (51%) | 36 (51%) | .990 | 50 (44%) | 30 (35%) | .196 |
| - financial problems (some/a lot) | 7 (8%) | 6 (9%) | .997 | 6 (5%) | 3 (3%) | .546 |
| - perceived support (some/a lot) | 52 (63%) | 37 (52%) | .187 | 84 (73%) | 56 (64%) | .186 |
| - physical health problems (some/a lot) | 52 (63%) | 46 (65%) | .783 | 48 (42%) | 31 (36%) | .378 |
| CG = caregiver, CR = care recipient  ^a^ Median (interquartile range) | | | | | | |

| Table S2A: Baseline univariate and multivariate logistic regression analyses, spousal caregivers (N=154) | | | | | | | | | | | | |
| --- | --- | --- | --- | --- | --- | --- | --- | --- | --- | --- | --- | --- |
|  | Fulfilment | | | | | | Relational problems | | | | | |
|  | Univariate results | | | Multivariate results | | | Univariate results | | | Multivariate results | | |
| **Spousal caregivers (N=154)** | OR | 95% CI | p | OR | 95% CI | p | OR | 95% CI | p | OR | 95% CI | p |
| Covariates^a^ |  |  |  |  |  |  |  |  |  |  |  |  |
| - Age CG | 1.007 | (.960-1.056) | .772 |  |  |  | 1.016 | (.967-1.067) | .533 |  |  |  |
| - Female CG | 1.045 | (.546-2.002) | .894 |  |  |  | **2.212** | (1.106-4.424) | .025 | **2.402** | (1.164-4.957) | .018 |
| - Informal support available | 1.005 | (.487-2.076) | .989 |  |  |  | 1.705 | (.824-3.528) | .151 |  |  |  |
| - Self-rated health CG | 1.212 | (.856-1.717) | .279 |  |  |  | .753 | (.523-1.084) | .127 |  |  |  |
| Time investment – hours a week |  |  |  |  |  |  |  |  |  |  |  |  |
| - Household care tasks | 1.007 | (.987-1.028) | .501 |  |  |  | .995 | (.975-1.016) | .641 |  |  |  |
| - Personal care tasks | 1.038 | (.994-1.084) | .092 | 1.038 | (.994-1.084) | .092 | .996 | (.964-1.030) | .826 |  |  |  |
| - Practical care tasks | 1.029 | (.981-1.079) | .244 |  |  |  | 1.011 | (.969-1.054) | .624 |  |  |  |
| Health situation CR |  |  |  |  |  |  |  |  |  |  |  |  |
| - Multimorbidity | 1.021 | (.856-1.219) | .816 |  |  |  | 1.137 | (.950-1.360) | .162 |  |  |  |
| - Functional limitations | 1.030 | (.953-1.114) | .450 |  |  |  | **1.109** | (1.024-1.201) | .011 | **1.113**^c^ | (1.027-1.207) | .009 |
| - Cognitive functioning problems^b^ | .744 | (.391-1.417) | .368 |  |  |  | **2.619** | (1.325-5.178) | .006 | **2.766**^c^ | (1.376-5.558) | .004 |
| Nagelkerke R2 |  |  |  | .032 |  |  |  |  |  | .138 |  |  |
| CG = caregiver, CR = care recipient, OR=odds ratio, 95% CI= 95% confidence interval. Bold OR’s are significant with p < .05.  Note. The subjective burden dimensions financial problems could not be examined, because of low numbers of spousal caregivers with some or severe financial problems (N=13)  ^a^ Care recipient’s institutionalization was excluded, because of low numbers of cases in the combination with burden dimensions (e.g. 2 spousal caregivers with institutionalized care recipient and no relational problems)  ^b^ Cognitive functioning problems was dichotomized (no vs. some/severe), because of low numbers of cases in the combination with burden dimensions (e.g. 1 caregiver with severe cognitive functioning problems care recipient and no physical health problems)  ^c^ Results from separate regression models, because functional limitations and cognitive functioning problems were collinear. | | | | | | | | | | | | |

| Table S2B: Baseline univariate and multivariate logistic regression analyses, spousal caregivers (N=154) | | | | | | | | | | | | |
| --- | --- | --- | --- | --- | --- | --- | --- | --- | --- | --- | --- | --- |
|  | Mental health problems | | | | | | Daily activities problems | | | | | |
|  | Univariate results | | | Multivariate results | | | Univariate results | | | Multivariate results | | |
| **Spousal caregivers (N=154)** | OR | 95% CI | p | OR | 95% CI | p | OR | 95% CI | p | OR | 95% CI | p |
| Covariates^a^ |  |  |  |  |  |  |  |  |  |  |  |  |
| - Age CG | .992 | (.947-1.040) | .738 |  |  |  | 1.011 | (.964-1.059) | .655 |  |  |  |
| - Female CG | **2.135** | (1.110-4.104) | .023 | **2.153** | (1.031-4.498) | .041 | **2.500** | (1.294-4.831) | .006 | **2.749** | (1.322-5.717) | .007 |
| - Informal support available | 1.396 | (.680-2.865) | .363 |  |  |  | 1.347 | (.657-2.765) | .416 |  |  |  |
| - Self-rated health CG | **.523** | (.359-.762) | .001 | **.523** | (.348-.785) | .002 | **.564** | (.391-.814) | .002 | **.543** | (.365-.802) | .003 |
| Time investment – hours a week |  |  |  |  |  |  |  |  |  |  |  |  |
| - Household care tasks | 1.006 | (.986-1.026) | .549 |  |  |  | .998 | (.979-1.018) | .868 |  |  |  |
| - Personal care tasks | 1.032 | (.994-1.072) | .102 |  |  |  | **1.043** | (1.000-1.088) | .048 | 1.018 | (.974-1.065) | .427 |
| - Practical care tasks | 1.008 | (.967-1.051) | .697 |  |  |  | 1.027 | (.982-1.075) | .243 |  |  |  |
| Health situation CR |  |  |  |  |  |  |  |  |  |  |  |  |
| - Multimorbidity | **1.459** | (1.186-1.794) | .000 | **1.305** | (1.035-1.645) | .024 | 1.181 | (.985-1.416) | .073 | 1.056^c^ | (.857-1.300) | .609 |
| - Functional limitations | **1.162** | (1.067-1.265) | .001 | **1.142**^c^ | (1.039-1.256) | .006 | **1.150** | (1.057-1.251) | .001 | **1.137**^c^ | (1.023-1.263) | .017 |
| - Cognitive functioning problems^b^ | 1.877 | (.989-3.559) | .054 | 1.359^c^ | (.665-2.776) | .400 | **1.776** | (.938-3.636) | .078 | 1.047 | (.483-2.272) | .907 |
| Nagelkerke R2 |  |  |  | .284 |  |  |  |  |  | .239 |  |  |
| CG = caregiver, CR = care recipient, OR=odds ratio, 95% CI= 95% confidence interval. Bold OR’s are significant with p < .05.  Note. The subjective burden dimensions financial problems could not be examined, because of low numbers of spousal caregivers with some or severe financial problems (N=13)  ^a^ Care recipient’s institutionalization was excluded, because of low numbers of cases in the combination with burden dimensions (e.g. 2 spousal caregivers with institutionalized care recipient and no relational problems)  ^b^ Cognitive functioning problems was dichotomized (no vs. some/severe), because of low numbers of cases in the combination with burden dimensions (e.g. 1 caregiver with severe cognitive functioning problems care recipient and no physical health problems)  ^c^ Results from separate regression models, because multimorbidity, functional limitations, and cognitive functioning problems were collinear. | | | | | | | | | | | | |

| Table S2C: Baseline univariate and multivariate logistic regression analyses, spousal caregivers (N=154) | | | | | | | | | | | | |
| --- | --- | --- | --- | --- | --- | --- | --- | --- | --- | --- | --- | --- |
|  | Perceived social support | | | | | | Physical health problems | | | | | |
|  | Univariate results | | | Multivariate results | | | Univariate results | | | Multivariate results | | |
| **Spousal caregivers (N=154)** | OR | 95% CI | p | OR | 95% CI | p | OR | 95% CI | p | OR | 95% CI | p |
| Covariates^a^ |  |  |  |  |  |  |  |  |  |  |  |  |
| - Age CG | 1.043 | (.994-1.095) | .088 | 1.015 | (.963-1.070) | .578 | 1.010 | (.962-1.060) | .701 |  |  |  |
| - Female CG | 1.387 | (.725-2.652) | .323 |  |  |  | 1.368 | (.704-2.658) | .355 |  |  |  |
| - Informal support available | **4.199** | (1.784-9.882) | .001 | **3.279** | (1.327-8.103) | .010 | 1.805 | (.822-3.964) | .141 |  |  |  |
| - Self-rated health CG | **.700** | (.496-.990) | .043 | .758 | (.525-1.094) | .139 | ^c^ |  |  |  |  |  |
| Time investment – hours a week |  |  |  |  |  |  |  |  |  |  |  |  |
| - Household care tasks | 1.015 | (.994-1.037) | .165 |  |  |  | 1.005 | (.984-1.026) | .636 |  |  |  |
| - Personal care tasks | 1.021 | (.984-1.059) | .269 |  |  |  | .998 | (.966-1.031) | .890 |  |  |  |
| - Practical care tasks | 1.024 | (.978-1.073) | .312 |  |  |  | 1.024 | (.975-1.075) | .340 |  |  |  |
| Health situation CR |  |  |  |  |  |  |  |  |  |  |  |  |
| - Multimorbidity | 1.169 | (.971-1.408) | .099 | 1.094 | (.887-1.349) | .401 | 1.172 | (.966-1.423) | .108 |  |  |  |
| - Functional limitations | **1.119** | (1.028-1.218) | .009 | 1.069 | (.975-1.173) | .156 | **1.126** | (1.030-1.231) | .009 | 1.097 | (.997-1.208) | .058 |
| - Cognitive functioning problems^b^ | 1.496 | (.787-2.844) | .220 |  |  |  | **2.241** | (1.146-4.382) | .018 | 1.681 | (.811-3.4875) | .163 |
| Nagelkerke R2 |  |  |  | .165 |  |  |  |  |  | .082 |  |  |
| CG = caregiver, CR = care recipient, OR=odds ratio, 95% CI= 95% confidence interval. Bold OR’s are significant with p < .05.  Note. The subjective burden dimensions financial problems could not be examined, because of low numbers of spousal caregivers with some or severe financial problems (N=13)  ^a^ Care recipient’s institutionalization was excluded, because of low numbers of cases in the combination with burden dimensions (e.g. 2 spousal caregivers with institutionalized care recipient and no relational problems)  ^b^ Cognitive functioning problems was dichotomized (no vs. some/severe), because of low numbers of cases in the combination with burden dimensions (e.g. 1 caregiver with severe cognitive functioning problems care recipient and no physical health problems)  ^c^ Self-rated health CG was not included in the logistic regression analysis for physical health problems, because of overlap with physical health problems. | | | | | | | | | | | | |

| Table S3A: Baseline univariate and multivariate logistic regression analyses, adult-child caregivers (N=202) | | | | | | | | | | | | |
| --- | --- | --- | --- | --- | --- | --- | --- | --- | --- | --- | --- | --- |
|  | Fulfilment | | | | | | Relational problems | | | | | |
|  | Univariate results | | | Multivariate results | | | Univariate results | | | Multivariate results | | |
| **Adult-child caregivers (N=202)** | OR | 95% CI | p | OR | 95% CI | p | OR | 95% CI | p | OR | 95% CI | p |
| Covariates |  |  |  |  |  |  |  |  |  |  |  |  |
| - Age CG | 1.003 | (.965-1.042) | .874 |  |  |  | 1.020 | (.980-1.061) | .331 |  |  |  |
| - Female CG | 1.071 | (.559-2.054) | .835 |  |  |  | 1.093 | (.552-2.166) | .799 |  |  |  |
| - Informal support available | 1.468 | (.826-2.609) | .191 |  |  |  | 1.161 | (.644-2.093) | .619 |  |  |  |
| - Institutionalized CR | 1.462 | (.822-2.602) | .196 |  |  |  | .996 | (.547-1.816) | .990 |  |  |  |
| - Self-rated health CG | **1.399** | (1.044-1.875) | .025 | **1.399** | (1.044-1.875) | .025 | .857 | (.637-1.152) | .307 |  |  |  |
| Time investment – hours a week |  |  |  |  |  |  |  |  |  |  |  |  |
| - Household care tasks | 1.020 | (.969-1.074) | .448 |  |  |  | 1.019 | (.971-1.070) | .444 |  |  |  |
| - Personal care tasks | 1.056 | (.927-1.202) | .412 |  |  |  | 1.003 | (.898-1.120) | .959 |  |  |  |
| - Practical care tasks | 1.033 | (.963-1.108) | .367 |  |  |  | 1.036 | (.972-1.103) | .281 |  |  |  |
| Health situation CR |  |  |  |  |  |  |  |  |  |  |  |  |
| - Multimorbidity | 1.036 | (.901-1.192) | .618 |  |  |  | **1.203** | (1.039-1.392) | .013 | **1.166** | (1.005-1.353) | .042 |
| - Functional limitations | 1.001 | (.931-1.076) | .978 |  |  |  | 1.048 | (.972-1.131) | .222 |  |  |  |
| - Cognitive functioning problems^a^ | .625 | (.351-1.113) | .111 |  |  |  | **2.205** | (1.184-4.105) | .013 | **1.949** | (1.031-3.686) | .040 |
| Nagelkerke R2 |  |  |  | .035 |  |  |  |  |  | .071 |  |  |
| CG = caregiver, CR = care recipient, OR=odds ratio, 95% CI= 95% confidence interval. Bold OR’s are significant with p < .05.  Note.The subjective burden dimensions financial problems could not be examined, because of low numbers of adult-child caregivers with some or severe financial problems (N=9)  ^a^ Cognitive functioning problems was dichotomized (no vs. some/severe), because of low numbers of cases in the combination with burden dimensions (e.g. 1 caregiver with severe cognitive functioning problems care recipient and no physical health problems) | | | | | | | | | | | | |

| Table S3B: Baseline univariate and multivariate logistic regression analyses, adult-child caregivers (N=202) | | | | | | | | | | | | |
| --- | --- | --- | --- | --- | --- | --- | --- | --- | --- | --- | --- | --- |
|  | Mental health problems | | | | | | Daily activities problems | | | | | |
|  | Univariate results | | | Multivariate results | | | Univariate results | | | Multivariate results | | |
| **Adult-child caregivers (N=202)** | OR | 95% CI | p | OR | 95% CI | p | OR | 95% CI | p | OR | 95% CI | p |
| Covariates |  |  |  |  |  |  |  |  |  |  |  |  |
| - Age CG | .969 | (.931-1.009) | .127 |  |  |  | .970 | (.933-1.009) | .129 |  |  |  |
| - Female CG | **3.111** | (1.410-6.866) | .005 | **2.692** | (1.125-6.443) | .026 | 1.173 | (.604-2.280) | .637 |  |  |  |
| - Informal support available | 1.239 | (.692-2.220) | .471 |  |  |  | 1.306 | (.737-2.313) | .361 |  |  |  |
| - Institutionalized CR | **.444** | (.246-.805) | .007 | **.374** | (.187-.747) | .005 | .596 | (.334-1.065) | .081 | .471 | (.243-.914) | .026 |
| - Self-rated health CG | **.536** | (.385-.747) | .000 | **.514** | (.355-.744) | .000 | **.561** | (.409-.770) | .000 | **.539** | (.363-.798) | .000 |
| Time investment – hours a week |  |  |  |  |  |  |  |  |  |  |  |  |
| - Household care tasks | **1.075** | (1.017-1.137) | .011 | **1.074**^b^ | (1.011-1.140) | .020 | 1.053 | (.999-1.110) | .052 | 1.047^b^ | (.992-1.106) | .097 |
| - Personal care tasks | **1.279** | (1.063-1.540) | .009 | **1.298**^b^ | (1.064-1.583) | .010 | **1.194** | (1.010-1.412) | .038 | **1.199**^b^ | (1.009-1.425) | .040 |
| - Practical care tasks | 1.023 | (.961-1.089) | .469 |  |  |  | 1.038 | (.973-1.108) | .254 |  |  |  |
| Health situation CR |  |  |  |  |  |  |  |  |  |  |  |  |
| - Multimorbidity | 1.137 | (.986-1.311) | .077 | 1.147 | (.973-1.351) | .102 | **1.240** | (1.070-1.437) | .004 | **1.266** | (1.079-1.487) | .004 |
| - Functional limitations | 1.033 | (.959-1.114) | .391 |  |  |  | 1.028 | (.955-1.106) | .463 |  |  |  |
| - Cognitive functioning problems^a^ | 1.381 | (.764-2.499) | .285 |  |  |  | .989 | (.558-1.754) | .970 |  |  |  |
| Nagelkerke R2 |  |  |  | .277 |  |  |  |  |  | .323 |  |  |
| CG = caregiver, CR = care recipient, OR=odds ratio, 95% CI= 95% confidence interval. Bold OR’s are significant with p < .05.  Note.The subjective burden dimensions financial problems could not be examined, because of low numbers of adult-child caregivers with some or severe financial problems (N=9)  ^a^ Cognitive functioning problems was dichotomized (no vs. some/severe), because of low numbers of cases in the combination with burden dimensions (e.g. 1 caregiver with severe cognitive functioning problems care recipient and no physical health problems)  ^b^ Results from separate regression models, because household care tasks and personal care tasks were collinear. | | | | | | | | | | | | |

| Table S3C: Baseline univariate and multivariate logistic regression analyses, adult-child caregivers (N=202) | | | | | | | | | | | | |
| --- | --- | --- | --- | --- | --- | --- | --- | --- | --- | --- | --- | --- |
|  | Perceived social support | | | | | | Physical health problems | | | | | |
|  | Univariate results | | | Multivariate results | | | Univariate results | | | Multivariate results | | |
| **Adult-child caregivers (N=202)** | OR | 95% CI | p | OR | 95% CI | p | OR | 95% CI | p | OR | 95% CI | p |
| Covariates |  |  |  |  |  |  |  |  |  |  |  |  |
| - Age CG | .992 | (.952-1.033) | .694 |  |  |  | .995 | (.957-1.034) | .781 |  |  |  |
| - Female CG | 1.275 | (.644-2.525) | .486 |  |  |  | **2.764** | (1.315-5.810) | .007 | **2.800** | (1.280-6.124) | .010 |
| - Informal support available | **3.769** | (1.880-7.556) | .000 | **3.578** | (1.774-7.218) | .000 | .741 | (.415-1.323) | .311 |  |  |  |
| - Institutionalized CR | **.464** | (.240-.897) | .023 | .511 | (.258-1.011) | .054 | .818 | (.458-1.463) | .498 |  |  |  |
| - Self-rated health CG | .822 | (.610-1.108) | .198 |  |  |  | ^b^ |  |  |  |  |  |
| Time investment – hours a week |  |  |  |  |  |  |  |  |  |  |  |  |
| - Household care tasks | 1.027 | (.968-1.090) | .373 |  |  |  | 1.053 | (1.000-1.110) | .052 | **1.056**^c^ | (1.001-1.114) | .044 |
| - Personal care tasks | 1.087 | (.916-1.289) | .340 |  |  |  | **1.295** | (1.065-1.576) | .010 | **1.291**^c^ | (1.057-1.577) | .012 |
| - Practical care tasks | 1.033 | (.954-1.118) | .423 |  |  |  | **1.080** | (1.000-1.166) | .049 | 1.070^c^ | (.993-1.154) | .076 |
| Health situation CR |  |  |  |  |  |  |  |  |  |  |  |  |
| - Multimorbidity | .994 | (.857-1.152) | .934 |  |  |  | 1.148 | (.996-1.323) | .056 | 1.125 | (.968-1.308) | .125 |
| - Functional limitations | .976 | (.903-1.055) | .538 |  |  |  | 1.052 | (.977-1.132) | .182 |  |  |  |
| - Cognitive functioning problems^a^ | 1.273 | (.695-2.329) | .434 |  |  |  | .805 | (.454-1.427) | .457 |  |  |  |
| Nagelkerke R2 |  |  |  | .131 |  |  |  |  |  | .149 |  |  |
| CG = caregiver, CR = care recipient, OR=odds ratio, 95% CI= 95% confidence interval. Bold OR’s are significant with p < .05.  Note.The subjective burden dimensions financial problems could not be examined, because of low numbers of adult-child caregivers with some or severe financial problems (N=9)  ^a^ Cognitive functioning problems was dichotomized (no vs. some/severe), because of low numbers of cases in the combination with burden dimensions (e.g. 1 caregiver with severe cognitive functioning problems care recipient and no physical health problems)  ^a^ Self-rated health CG was not included in the logistic regression analysis for physical health problems, because of overlap with physical health problems..  ^c^ Results from separate regression models, because household care tasks, personal care tasks, and practical care tasks were collinear. | | | | | | | | | | | | |
